# Alkali-Activated Mortars with Recycled Fines and Hemp as a Sand

**DOI:** 10.3390/ma14164580

**Published:** 2021-08-15

**Authors:** Edyta Pawluczuk, Katarzyna Kalinowska-Wichrowska, Mahfooz Soomro

**Affiliations:** 1 Faculty of Civil Engineering and Environmental Sciences, Bialystok University of Technology, 15-351 Bialystok, Poland; e.pawluczuk@pb.edu.pl; 2 School of Engineering and Built Environment, Western Sydney University, Penrith, NSW 2751, Australia; m.soomro@westernsydney.edu.au

**Keywords:** recycled fines, hemp filler, alkali-activator, eco-friendly fillers, CO_2_ emissions, circular economy

## Abstract

Nowadays, effective and eco-friendly ways of using waste materials that could replace natural resources (for example, sand) in the production of concrete composites are highly sought. The article presents the results of research on geopolymer composites produced from two types of waste materials—hemp and fine fractions recovered from recycled cement concrete, which were both used as a replacement for standard sand. A total of two research experiments were conducted. In the first experiment, geopolymer mortars were made using the standard sand, which was substituted with recycled fines, from 0% to 30% by weight. In the second study, geopolymers containing organic filler were designed, where the variables were (i) the amount of hemp and the percent of sand by volume (0%, 2.5%, and 5%) and(ii) the amount of hydrated lime and the percent of fly ash (by weight) (0%, 2%, and 4%) that were prepared. In both cases, the basic properties of the prepared composites were determined, including their flexural strength, compressive strength, volume density in a dry and saturated state, and water absorption by weight. Observations of the microstructure of the geopolymers using an electron and optical microscope were also conducted. The test results show that both materials (hemp and recycled fines) and the appropriate selection of the proportions of mortar components and can produce composites with better physical and mechanical properties compared to mortars made of only natural sand. The detailed results show that recycled fines (RF) can be a valuable substitute for natural sand. The presence of 30% recycled fines (by weight) as a replacement for natural sand in the alkali-activated mortar increased its compressive strength by 26% and its flexural strength by 9% compared to control composites (compared to composites made entirely of sand without its alternatives). The good dispersion of both materials in the geopolymer matrix probably contributed to filling of the pores and reducing the water absorption of the composites. The use of hemp as a sand substitute generally caused a decrease in the strength properties of geopolymer mortar, but satisfactory results were achieved with the substitution of 2.5% hemp (by volume) as a replacement for standard sand (40 MPa for compressive strength, and 6.3MPa for flexural strength). Both of these waste materials could be used as a substitute for natural sand and are examples of an eco-friendly and sustainable substitution to save natural, non-renewable resources.

## 1. Introduction

During the cement production process, large amounts of carbon dioxide are emitted. The cement industry alone accounts for approximately 4.1% of the EU’s and around 8 to 10% of world’s anthropogenic CO_2_ emissions [1]. Carbon dioxide in cement production is emitted in two primary ways: the calcination of calcium carbonate and fuel combustion in the cement kiln as well as in two indirect ways: electrical energy consumption for running the process equipment in the cement plant and for the transportation of raw materials and cement [2,3]. Over time, strategies to reduce the CO_2_ emissions have been developed: improving production processes, modernizing equipment, replacing primary fuels with alternative fuels created from waste, optimizing the cement composition, and recovering heat energy from the production processes [4]. However, almost half of the CO_2_ is produced from the calcination process in the kiln where the conversion of raw materials takes place. It has been estimated that on average for cement plants around the world, CO_2_ emissions from the calcination process amount to nearly 50%, while the emissions from fuel combustion amount to about 40% of total emissions [3,5]. Therefore, it is beneficial for the environment to replace part of the cement clinker with supplementary cementitious materials, such as blast furnace slag [6], fly ash [7], and natural pozzolan [8], to significantly reduce the CO_2_ emissions.

It is estimated that the synthesis of alkali-activated binders is a much less energy-consuming process than that of the production of Portland cement and produces 4–8 times less carbon dioxide [9]. Regarding the terminology and difference between “geopolymers” and “alkali-activated binders”, Davidovits [10] mentions that alkali-activated materials cannot be called geopolymers; however, in the literature, both the terms have been used interchangeably. Therefore, in this article both “geopolymers” and “alkali-activated mortars” have also been used interchangeably. One of the main drivers of geopolymer technology is the possibility of creating a real alternative to cement concrete [10].

These materials can provide comparable performance to conventional concrete in a range of applications but with the added advantage of significantly reducing greenhouse (GHG) emissions [11]. For this reason, geopolymer is also called “green concrete”.

Research on the use of geopolymer composites in construction has been conducted for many years [12,13,14]. Sun et al. produced a geopolymer from the alkaline activation of ceramic waste using water glass and potassium hydroxide (KOH) as activators, and after 28 days, the specimens exhibited a compressive strength of 71 MPa [15]. However, it was necessary to heat cure the material at a temperature of 60 °C. Reig et al. [16] evaluated the influence of the alkaline activator concentration (NaOH and water glass) and the use of Ca(OH)_2_ on mortars made from sanitary porcelain waste.

The produced specimens showed a compressive strength of 36 MPa when cured at 65 °C for up to 7 days. According to Zaharaki et al. [17], the strength of alkali-activated specimens is affected by the molar concentration of the activating solution, i.e., NaOH. He deduced that the optimum NaOH molarity of around 10 M for the alkali activation of slag and C&D waste components.

However, at a high activator concentration, the workability of the geopolymer mixture is especially reduced in the presence of porous C&D waste. Allahverdi and Kani [18] developed a cement using the waste from a brick production plant and waste concrete that was activated with NaOH in the proportion of 8% Na_2_O content concerning the binder. Results obtained for 28-day compressive strength validated that waste brick was more suitable than waste concrete for geopolymerization. The maximum 28-day compressive strength achieved was 40 MPa for a system comprising only waste brick and containing 8% Na_2_O by weight with respect to the dry binder. The strength increased with the increase in Na_2_O percent. Research on geopolymer mortars containing NaOH/NaSi_2_O_3_ in the ratio of 2.5 was conducted by Abdullah et al. [19]. This is a non-ecological solution due to the higher content of harmful sodium hydroxide; hence, its use in the geopolymer should be limited.

A less optimistic approach to the issues of geopolymer production was presented by Habert et al. [20]. The authors agree that the production of such a binder has a slightly lower impact on global warming than conventional concrete, whilst having a higher environmental impact related to categories other than GHG gases. This is due to the energy consumption in the production of sodium silicate. Geopolymer concrete made from fly ash or granulated blast furnace slag requires less sodium silicate for activation than geopolymer concrete produced from pure metakaolin. However, when the production of fly ash and granulated blast furnace slag is taken into account during the life cycle assessment, it appears that geopolymer concrete has a similar impact on global warming as conventional concrete. As such, future research and development in the field of geopolymer concrete should focus on two potential solutions. First, the use of industrial waste that is not recyclable within other industries, and second, the production of geopolymer concrete should use a mix of blast furnace slag and activated clays. In addition, the production of geopolymer concrete should aim to minimize the amount of sodium silicate solution that is used. Furthermore, geopolymer composite production would gain from using waste material with a suitable Si/Al molar ratio in order to minimize the amount of sodium silicate solution used.

The aim of this research was to indicate that the geopolymer mortar produced from recycled fines and hemp has comparable properties to cement mortar and is a more ecological and environmentally friendly material. The second aspect was the possibility of managing, on the one hand, waste material in the form of a recycled fine fraction, and on the other, hemp as substitutes for natural sand in the mortar. The recycled fine is a by-product resulting from the production of high-quality recycled aggregate (according to the authors’ patented method [21,22]). As a result of the thermo-mechanical treatment of concrete rubble, up to 60% of the fine fraction (0–4 mm) is obtainable and can be used effectively with some additional care. Previously, the use of recycled fines in concrete was not recommended due to its unfavorable effects on the properties of fresh mix and the hardened composite [23]. However, during the last decade, the use of fine recycled aggregate has become more important in concrete production because of economic implications related to the shortage of natural sands commonly used in concrete production.

However, in the presence of fine recycled aggregate, worse concrete properties or those that were comparable to the control were obtained [24,25,26]. During the thermal-mechanical treatment of concrete rubble at the proposed temperature of 650 °C, all of the impurities present in the rubble are burnt, thermally activating the recycled fines, and the material partially regains its binding properties.

Hemp is one of the fastest growing plants on Earth and can be grown repeatedly. So far, hemp or chips have mainly been used for lightweight concretes as organic fillers to improve the insulation parameters of composites [27,28,29]. In the presented research, a new use for hemp was found as a replacement for sand in alkaline-activated mortars. The use of granulated blast furnace slag eliminates the need to heat-cure the geopolymer mortar, which is usually necessary to stimulate the hardening process. This study may contribute to the reduction of the consumption of non-renewable natural raw materials such as sand and the management of waste such as recycled fines resulting from the processing of C&D waste. Such activities may contribute to the protection of the natural environment. According to the authors, this can be a positive contribution to the future of the construction industry.

## 2. Materials and Methods

### 2.1. Characteristics of the Raw Materials

The fly ash (FA) that was used met the requirements of the standard EN 450-1:2012 “Fly ash for concrete—Part 1: Definitions, specifications, and compliance criteria”. It was a very fine-grained powder that was obtained from an electricity-producing plant in Poland. The chemical composition of the FA is presented in Table 1. The ground granulated blast furnace slag (GGBS) was obtained from a Polish steelwork; its chemical composition is also shown in Table 1.

Hydrated lime was used to improve the setting time and the early strength of the geopolymer mortar. The chemical composition of hydrated lime, ground granulated blast furnace slag (GGBS), and recycled fines (RF) are presented in Table 1.

**Figure 1:**
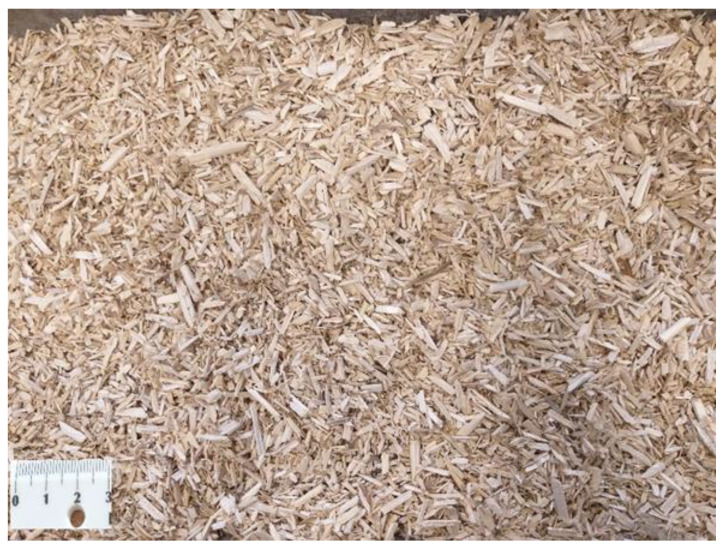
Hemp shives used in the preparation of geopolymer mortar.

Standard sand with a 0–2 mm particle size was used to prepare the samples. Hemp (organic materials) was used as a filler to prepare the geopolymer specimens. Hemp is a perennial crop that grows rapidly. It is a member of the family “Cannabaceae” in the order “Urticales” (which includes in the nettle family). In northern Europe, a hemp plant may reach a height of 1.5–4 m, whereas it can reach up to 10 m in height further south. It can be refined into a variety of commercial items, including paper, clothing, textiles, rope, biodegradable plastics, paint, insulation, and animal feed. Hemp fibres have high tensile strength and are therefore advantageous for use in concrete and geopolymer products [30]. The hemp used in this research is shown in Figure 1, and its main properties are presented in Table 2.

The alkaline activator used to prepare the samples was an aqueous solution of sodium silicate and sodium hydroxide of an 8 M concentration that was used in both experiments. The mass ratio of sodium silicate (Na_2_SiO_3_) to sodium hydroxide (NaOH) was 1.0. NaOH was selected based on studies by other researchers who showed that NaOH produced higher compressive strength compared to KOH [31]. The activator was added to the mixture in the amount of 60% of the binder weight to maintain the workability of the fresh mortar.

### 2.2. Research Methods

The bulk density of the hemp and the recycled fines were conducted according to EN 1097-3: 2000. The skeletal density of the RF and the water absorption of hemp tests were performed according to EN 1097-6: 2013. For the water absorption test, the hemp was placed in nets and was weighed for immersion in water. The humidity was determined from the different weights of the hemp in its natural state and when it was dried to a constant weight.

**Figure 2:**
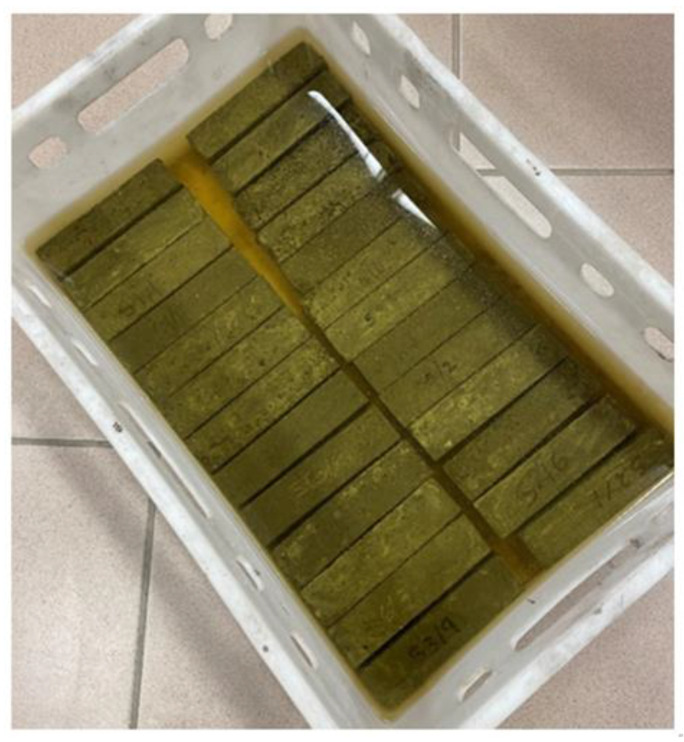
Samples 40 mm × 40 mm × 160 mm during saturation.

The geopolymer mortar specimens (40 mm × 40 mm × 160 mm) were prepared in accordance with EN 196-1: 2016 (Methods of testing cement. Determination of strength). The flexural and compressive strength tests were performed according to EN 196-1: 2016. The water absorbtion test was performed by determining the percentage increase in the weight of the specimens when they saturated with water in relation to the weight of the specimen in the dry state. After 28 days of curing, three samples from each series were selected for water absorbtion tests. The samples were first weighed to determine their initial mass, and they were then placed in an oven maintained at a temperature of 80 °C to dry until a constant weight was achieved. The samples were then immersed in water until a constant weight was obtained (Figure 2).

The volume density in a dry state and in a saturated state were determined based on EN 1015-10:1999 (Methods of test for mortar for masonry—Part 10: Determination of dry bulk density of hardened mortar).

### 2.3. The Technology of Recycling Cement Mortar to Recycled Fines

**Figure 3:**
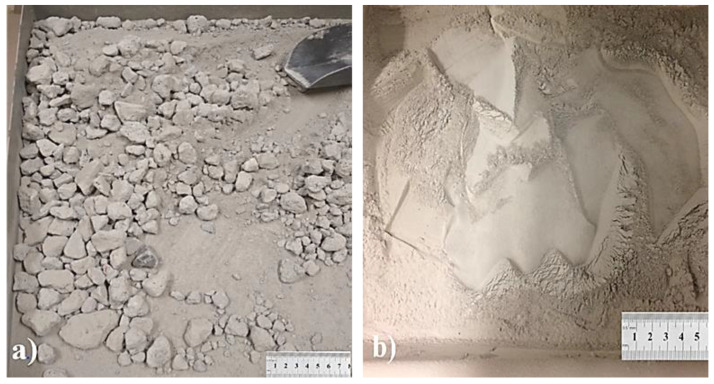
(**a**) Concrete rubble after thermo-mechanical treatment and (**b**) recycled fines after grinding.

To produce recycled fines for use in the experiment, debris from crushing 5-year-old C35/C45 concrete road curbs were used, and the recycled fines were obtained based on the thermo-mechanical treatment of concrete rubble according to the patent PAT.229887 Method [21]. First, the concrete elements were crushed to <4 cm dimensions in a laboratory jaw crusher. The crushed material was then calcined at 650 °C for about 60 min in a chamber furnace to weaken the bonding in the concrete mortar. After cooling, the heat-treated material was machined in a Los Angeles drum containing 3 steel balls for 500 revolutions (Figure 3a). The thermo-mechanical treatment method of the concrete rubble is described in [22]. The material was then sieved through a 4 mm sieve to obtain fine fractions, which were ground to a grain size of <0.125 mm and were used for further tests (Figure 3b).

**Figure 4:**
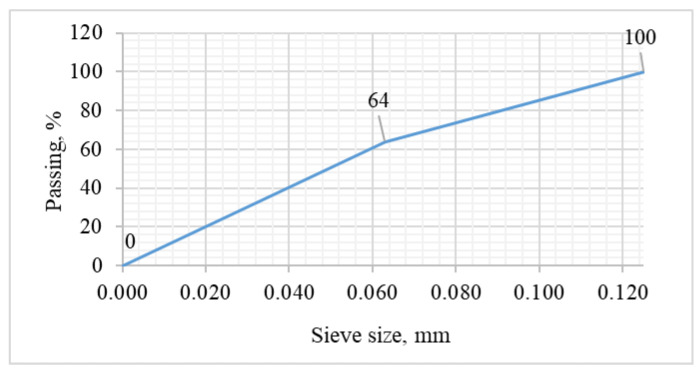
Sieve analysis curve for recycled fines; 0.125 mm size.

As it can be seen from Table 3, the bulk density of the recycled fines increased by 27% as a result of compaction. Figure 4 shows the sieving curve of the tested recycled fines.

**Figure 5:**
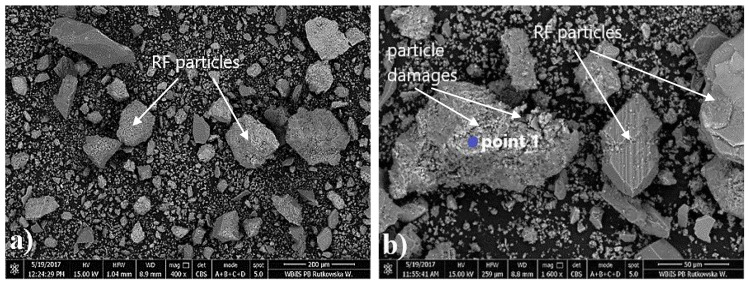
SEM observations of recycled fines calcined at 650 °C (Magnification: (**a**) left: ×400, (**b**) right: ×1600).

**Figure 6:**
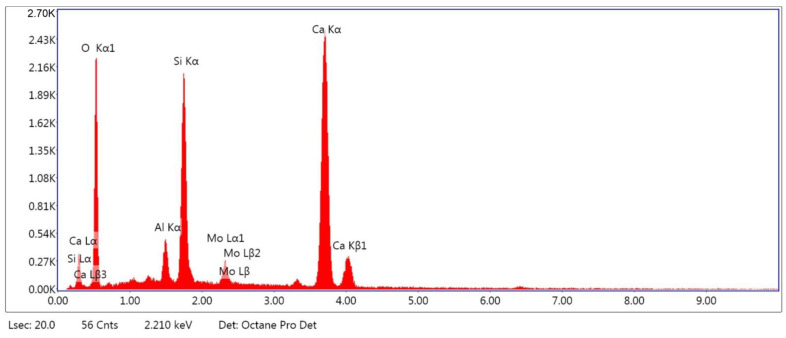
EDS element content analysis in recycled fines shown in Figure 5b.

As shown by the test results in Figure 4, the content of the powder fraction (<0.063 mm) in the tested recycled fines was approximately 64%. Figure 5 and Figure 6 show the SEM micrographs of the recycled fines calcined at a temperature of 650 °C and the electron-dispersive spectroscopy (EDS) element analysis, respectively. EDS provides the possibility of chemical composition analysis in very small and localized areas. The results of the elemental composition analysis are presented in Table 4.

Observations show that the recycled fines contain irregularly shaped particles with a rough surface (Figure 5a). The visible particle damage (Figure 5b) is the result of the thermal and mechanical treatment of the concrete rubble.

The analysis shows that the calcined recycled fines (RF) mainly consist of the CaO resulting from the high-temperature dehydration of Ca(OH)_2_ (portlandite), which is available in the recycled mortars, and SiO_2_, which is the main component of the sand (used in making concrete mortar) but that is also available in CSH gel. This is validated by the chemical composition of the RF presented in Table 1.

### 2.4. Experimental Design

There were two sets of experiments that were conducted. This first set of experiments used the geopolymer mortar with a recycled fines, and the second set of experiments were conducted using the geopolymer mortar with hemp.

#### 2.4.1. Selection of Variables and Development of the Experimental Plan—With Recycled Fines

An experiment based on one variable (X) was planned in order to investigate the influence of the contents of the recycled fines on the selected properties of geopolymer mortars. The percent variation of its contents is shown in Table 5.

Based on the above four percent contents (4 series), an experimental plan was established. In the experiment, the compressive strength, flexural strength, dry and saturated bulk density, and water absorption were tested. Table 6 shows the composition of the geopolymer mortars depending on the percentage of the recycled fines. The initial amounts of the components were assumed based on the composition of standard cement mortars. The composition of the mixes was designed with a constant amount of fly ash, alkaline activator, and activator/fly ash ratio. Standard sand was replaced with recycled fines in amounts ranging from 0% to 30% by weight.

After casting, the samples were placed in a laboratory oven for 24 h at 60 °C. The samples were heat cured was to accelerate the mortar hardening process. After demolding, the samples were cured in air-dry conditions. After 28 days, the samples were tested for their physical and mechanical properties.

#### 2.4.2. Selection of Variables and Development of the Experimental Plan—With Hemp

To better understand the relationship between factors such as hemp and lime and the properties of geopolymer mortar, an experiment was conducted based on two variables: *X*_1_-amount of hemp and *X*_2_-amount of lime. The detailed range of variability and the levels of the analyzed factors are presented in Table 7.

Table 8 shows the percentage distribution of the variable as per the series number. A total of nine different series of samples were prepared.

The initial composition of the geopolymer concrete mixture was adopted in the tests conducted in the laboratory and that were based on the literature. However, in the initial research plan, the first series that comprised hemp was quite dry and had minimal workability due to the hemp absorbing the moisture from the activator; hence, the composite was modified by adding the activator to the hemp before adding it to the mortar. Table 9 shows the composition of the geopolymer specimens by series.

**Figure 7:**
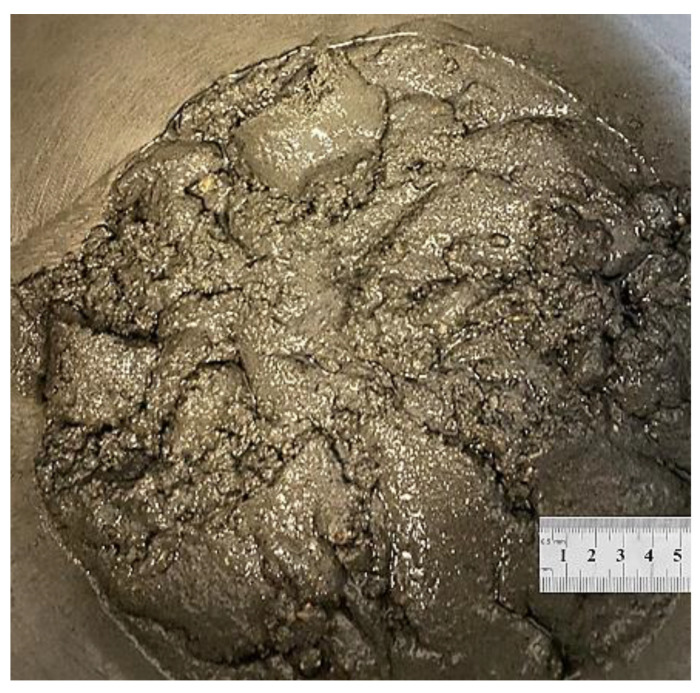
Geopolymer mortar after mixing.

Sand and the remaining dry ingredients were added first. Finally, an 8 M alkaline activator solution of NaOH was added to the mortar. Figure 7 depicts the geopolymer mortar after mixing.

The geopolymer mortar was compacted by vibrating it for 60 s in two layers. After 24 h, the samples were demolded and were placed in a closed container pending testing. Due to the presence of GGBS, heat curing at elevated temperature was not necessary to activate the hardening process, which represents a more ecological solution and reduces the cost of implementation.

## 3. Results and Discussion

### 3.1. Test Results of Geopolymer Mortar with Recycled Fines

**Figure 8:**
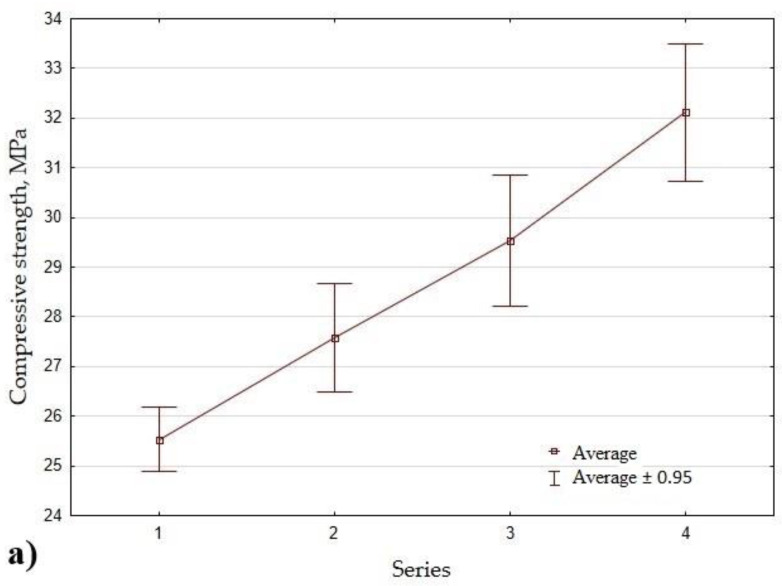

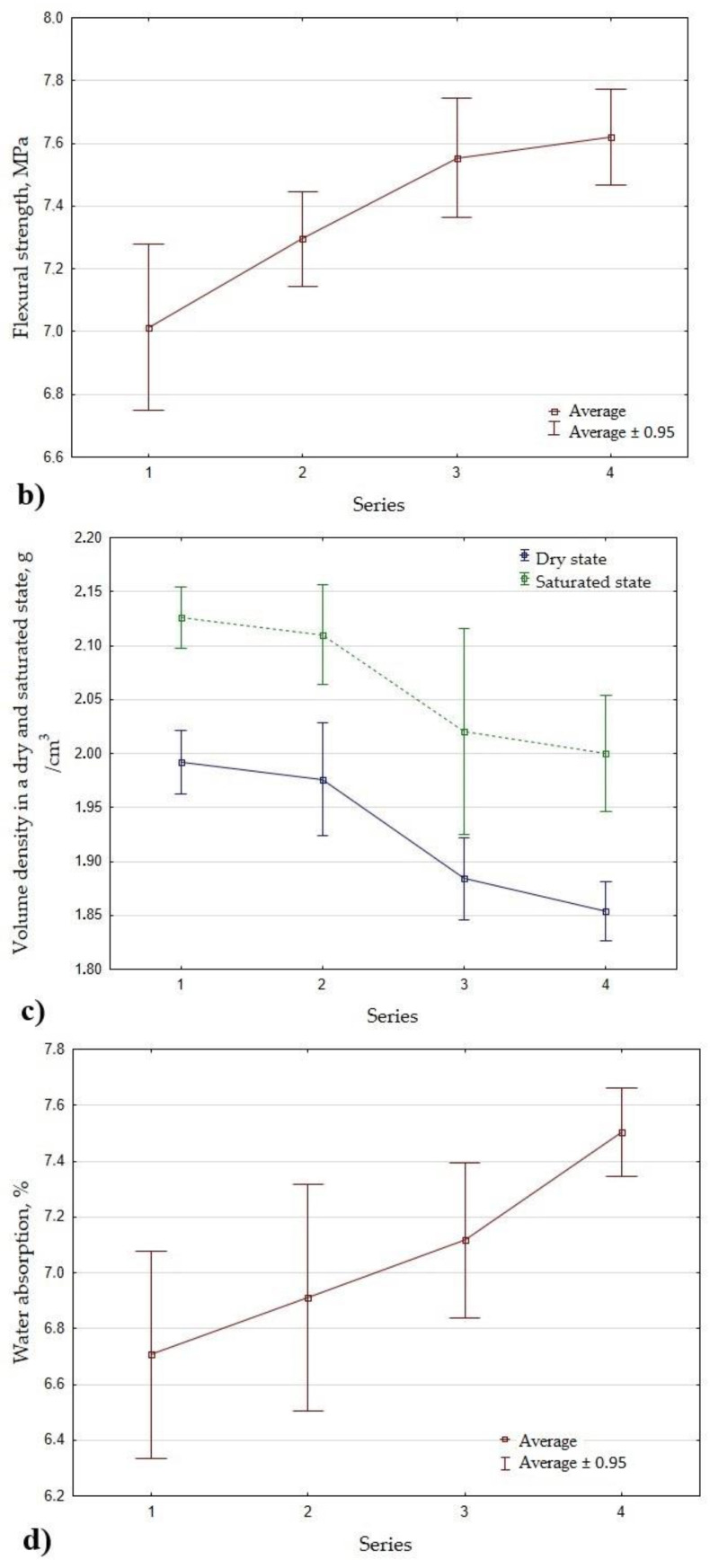
The average test results of geopolymer mortar containing recycled fines: (**a**) compressive strength after 28 days; (**b**) flexural strength after 28 days; (**c**) volume density in dry and saturated states; (**d**) the water absorption.

Figure 8 shows the average results of the properties of the geopolymer mortar containing RF for the individual series of the experiment. The compressive strength, flexural strength, volume density in a dry and saturated, and water absorption after 28 days of curing have been plotted in Figure 8a–d.

**Figure 9:**
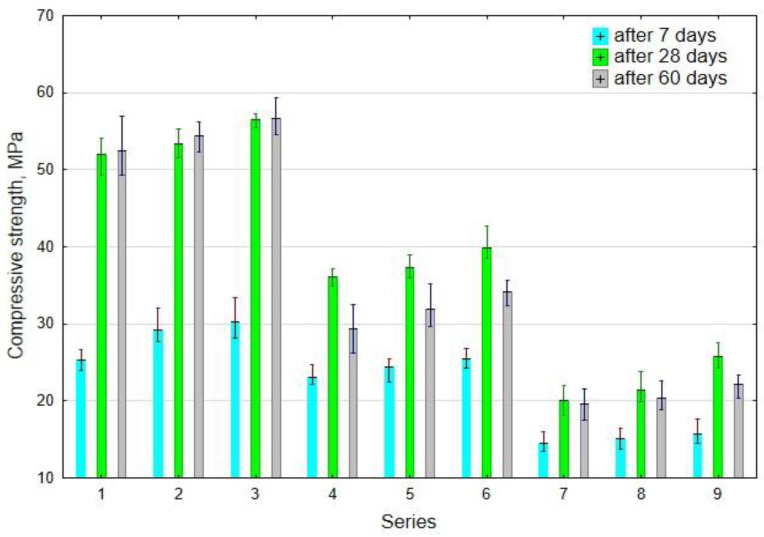
The average test results of the compressive strength of geopolymer mortars containing hemp after 7, 28, and 60 days.

Figure 8a shows a gradual increase in the compressive strength of the composites with the increase in the content of the recycled fines as a replacement for standard sand. When replacing 30% of the sand with RF, the strength increased by as much as 26% compared to Series 1. A similar beneficial effect of the presence of RF in the geopolymer mortar was noted in the case of flexural strength (Figure 8b and Figure 9). By increasing the RF content to 10%, 20%, and 30% of the sand (by weight) as a sand replacement, a 4%, 8%, and 9% increase in the flexural strength was obtained, respectively. In the fraction of recycled fine rich in CaO and SiO_2_ (resulting from the dehydration of Ca (OH)_2_, CSH gel, and Ettringite), the CSH gel and Ca(OH)_2_ can favor the formation of a new CSH gel in the geopolymer mortar.

**Figure 10:**
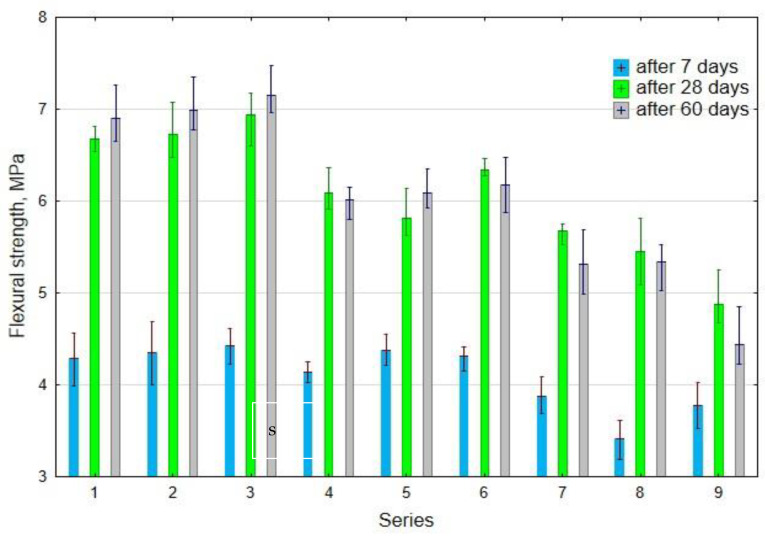
The average test results of the flexural strength of geopolymer mortars with hemp after 7, 28, and 60 days.

The changes in volume density were insignificant, as they did not exceed 7% and 6% in the dry and saturated state, respectively, when containing 30% RF (Figure 8c and Figure 10). Despite the density of the recycled fine being close to that of standard sand (Table 3), the RF particles were characterized by sharp edges, which made it difficult to pack them tightly in a geopolymer mortar. This is in line with the test results of other researchers, in which the overall shape of the particles is the dominant parameter condition in packing, and spherical beads have a display higher degree of packing than different tested sands compared to particles with sharp edges. [32].

**Figure 11:**
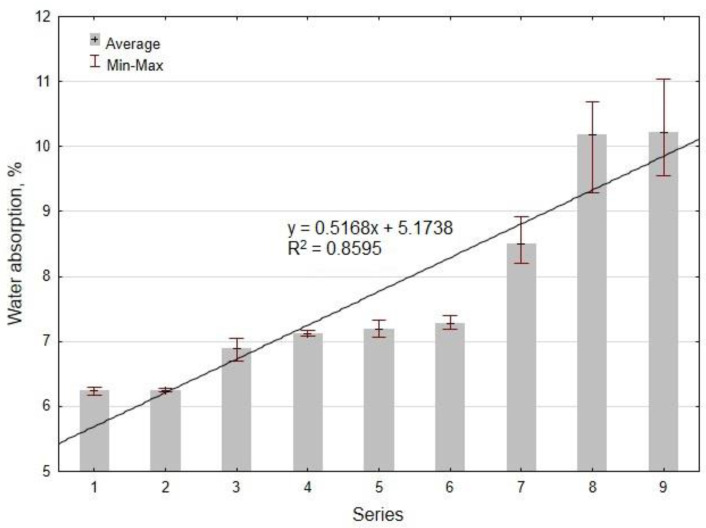
The average test results of the water absorption of the geopolymer mortars with hemp.

The recycled fine was characterized by higher water absorption than sand because it mainly consisted of somewhat porous cement paste. Therefore, when forming the samples, a partial reduction in the workability of the fresh mortar in the presence of RF was observed. This hindered the compaction of the mortar in the mold, thereby increasing its porosity. This is consistent with other results where, as the packing density increased, its porosity decreased, and the amount of water needed to fill the porosity was reduced, and more water became available to keep the mortar flowing [33]. Therefore, with the increase in the amount of RF, a slight increase in the water absorption of the mortar was noted from 6.71% (in the 1st series) to 7.50% (in the 4th series), as depicted in Figure 11 and Figure 8d. In the opinion of other researchers, the molarity of sodium hydroxide solution has an insignificant role in concrete water absorption—only a concentration in the range of 12 M to 18 M results in an improvement of this higher water absorption parameter [34]. In the experiment, a relatively low concentration of NaOH (8 M) was used. (8 M). The above results show that RF can be a valuable replacement for sand in alkaline-activated mortars, favorably affecting enhancing its strength properties.

### 3.2. Test Results of Geopolymer Mortar with Hemp

#### 3.2.1. Compressive Strength of Mortars

The average test results of the compressive strength for each series after 7, 28, and 60 days of curing have been plotted in Figure 9.

Based on the data in Figure 9, it can be concluded that the average compressive strength of the samples after 7 days of curing gradually declined with an increase in the hemp. With an increase in the hemp content from 0 to 5% as sand replacement by volume, a decrease in compressive strength was observed after 7 days of curing by 42% and 48% and the lime content of 0% and 4%, respectively. Increasing the lime content in the composite to 4% of the FA (by mass) had a positive effect on the improvement of compressive strength, showing an increase of 7% to 20% with the hemp content of 0% and 5%, respectively.

The compressive strength results after 28 days of curing exhibited a similar trend to that of 7 days of curing. However, there was a significant increase in the compressive strength after 28 days. The highest results were obtained in Series 3 at 56.5 MPa, which were observed with 4% lime content, which were 86% higher than the compressive strength of Series 3 after 7 days. Ca^+^ ions played a key role in the geopolymer matrix. In fact, a higher content helps for quicker geopolymerisation and the development of semi-crystalline Ca-Al-Si gel [35]. The beneficial effect of the presence of lime in an amount of up to 10% by weight of fly ash on the compressive strength of geopolymer mortars was also observed by other researchers [36]. The lowest strength was noticed in Series 7 with 5% hemp content. Mastali et al. [37] found that the addition of hemp fibres leads to a reduction of compressive strength because the fibres increase the porosity. This finding was also validated by other authors who studied the performance of composites containing hemp fibres [38].

Generally, the highest, about 90%, increase in compressive strength after 28 days, compared to 7 days strength, was observed in the series without hemp.

This finding is not comparable to the results presented by other authors, who observed an over 90% increase in the strength of 28-day strength compared to the strength obtained after 3–7 days [39,40]. In the presence of hemp, the increase in strength was about 50%. There was a 9% and 28% increase in compressive strength after 28 days with an increase in the amount of lime to 4% and 5% in the samples without hemp, respectively.

The samples without hemp (Series 1–3) only showed a slight increase in the compressive strength after 60 days compared to 28 days of curing. On the other hand, for the series with hemp (Series 4–9), an approximately 10% decrease (on average) was noticed after 60 days compared to 28 days of curing. The decrease in compressive strength after 60 days may have resulted due to the lack of protection of the hemp by impregnation, as in the case with cement composites [41,42]. The high absorbability of the hemp could have caused moisture absorption from the mortar, resulting in an increase in its volume and visible swelling of the samples over time. As research continues, it will be necessary to determine the best way to protect hemp by chemically coating/impregnating it.

#### 3.2.2. Flexural Strength of Mortars

The average results of the flexural strength tests for each series after 7, 28, and 60 days of curing, respectively, are presented in Figure 10.

As it can be seen from Figure 10, the highest flexural strength after 7 days was obtained for Series 3 (4.44 MPa) without hemp. A downward trend in flexural strength was observed with increasing hemp content. At 2.5% of its content, the decrease was slight and amounted to approximately 3–4%, while in the case of a 5% addition of hemp, the strength decreased in the range of 11–21%, depending on the amount of lime that had been added. The presence of lime in the geopolymer mortar did not cause any significant change in the flexural strength after 7 days. With the increase in the amount of lime to 4%, an increase in strength of 3% and 4% was observed in the samples without hemp and with 2.5% hemp, respectively. In the presence of 5% hemp, the presence of even a slight amount of lime reduced the flexural strength (by less than 2%), but this may be within the measurement error. This is not comparable to the results of studies presented by other authors, where the addition of lime of up to 10% or even 15% showed improved compressive as well as flexural strength [36,43]. According to these researchers, the addition of more than 15% lime shows a decreasing trend of workability, while in this study, the addition of a mere 5% of lime deteriorated the workability of the mortar.

After 28 days, similar to after 7 days, there was a downward trend in the flexural strength with the increase in the content in the geopolymer mortar. With 2.5% hemp content, a decrease in strength by an average of 10% was observed, but with 5% hemp content, there was a decrease of up to 30%. The unfavorable effect from the presence of lime on the flexural strength (decrease of 14%) was observed in the series with the highest hemp content. The greatest increase in flexural strength after 28 days, compared to 7 days, was shown by the samples without hemp, which amounted to approximately 55%. This tendency was similar to that of the compressive strength. In the presence of hemp, the increase in flexural strength was over 40%.

After 60 days of curing, only the samples from hemp-free series showed a slight increase in flexural strength of about 3.5%. The other series, similar to the compressive strength, showed a decrease in the flexural strength of 2% and 6% with 2.5% and 5% hemp content, respectively. The reason for the decrease in flexural strength after 60 days is probably the same as that mentioned before and is likely due to the lack of the coating/impregnation of hemp.

#### 3.2.3. Water Absorption of Mortars

The average results of the water absorption tests for each series after 28 days of curing are shown in Figure 11.

It was noticed that the water absorption of the geopolymer composite increased almost linearly with the increase in the content of hemp and lime. This is confirmed by the suitable fit of the regression equation to the results equal to 86%. The increase in water absorption caused by the presence of lime was insignificant due to the particle size distribution being similar to that of fly ash. The main reason for the increase in water absorption from 6.2% to 10.2% was the presence of highly porous hemp, which absorbed water intensively. The water absorption of the hemp was over 400%, while the moisture content was only 1.6%. This means that the hemp was dry despite being stored under laboratory conditions in closed bags. Generally, it can be stated that in our study, the water absorption of the geopolymer mortar without hemp was lower compared to the test results of other researchers [44].

#### 3.2.4. Volume Density of Mortars in a Dry and Saturated State

**Figure 12:**
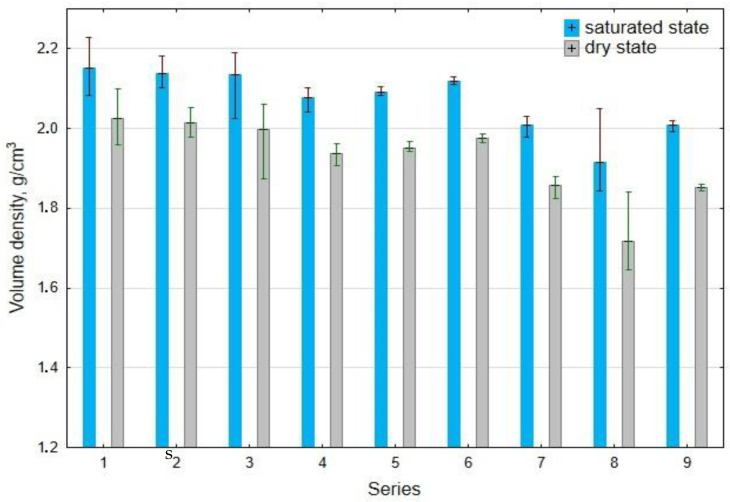
Average volume density of geopolymer mortars containing hemp in a dry and saturated state.

The average results of the volume density tests for each series in a dry and saturated state are shown in Figure 12.

When analyzing the results of the volume density of geopolymer mortars (Figure 12), an opposite water absorption trend was noticed. With the increase in the hemp content to 2.5% and 5%, the density of the composites decreased by 3% and 9%, respectively. This is of course due to the much lower density of hemp compared to sand. The phenomenon of lowering the density is advantageous due to the reduction of the weight of structural elements. For this reason, lightweight aggregates are used in concretes. It can be assumed that the composites with hemp should have better-insulating properties, but this will be the subject of further research. The volume density of the samples in a dry state was about 90–94% of the density in a saturated state.

#### 3.2.5. Optical Microscopy and Scanning Electron Microscopy (SEM) of Geopolymer Mortar with Hemp

**Figure 13:**
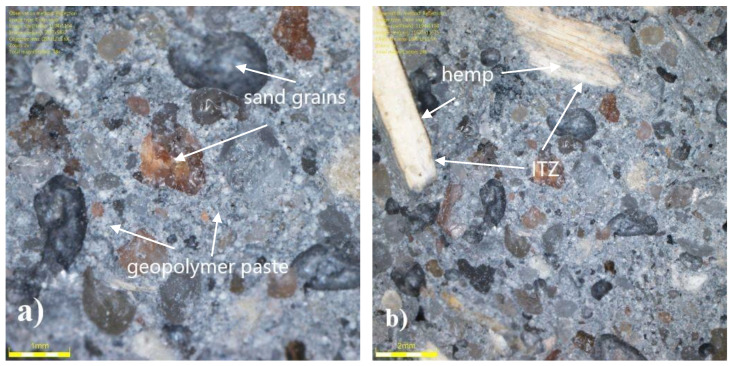
Optical microscopy observations of the structure of geopolymer mortar: (**a**) without hemp and (**b**) with hemp.

**Figure 14:**
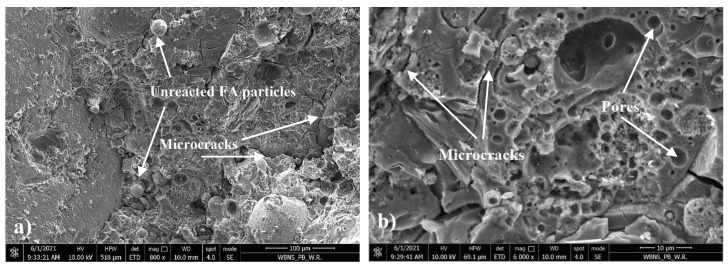
SEM micrographs of geopolymer mortar structure: (**a**) ×800 and (**b**) ×6000 magnification.

**Figure 15:**
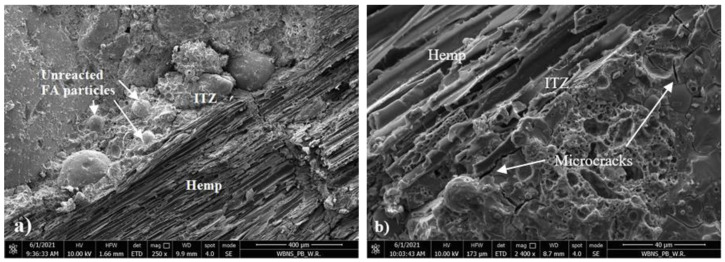
SEM micrographs of ITZ in geopolymer mortar containing hemp shives: (**a**) ×250 and (**b**) ×2400 magnification.

**Figure 16:**
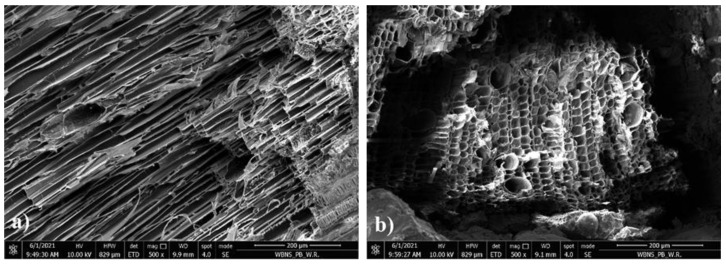
SEM micrographs of longitudinal and cross section of hemp shives: (**a**) ×500 and (**b**) ×500 magnification.

The cut through cross section of the sample after observing the flexural strength test using optical and scanning electron microscope was investigated. The micrographs are presented in Figure 13, Figure 14, Figure 15 and Figure 16.

Observations of the geopolymer mortar surface with an optical microscope (Figure 13a,b) indicated that the structure of the composite was quite compact with few visible pores. The hemp shives were tightly embedded in the mortar, which created a dense ITZ zone. No visible microcracks were observed.

In Figure 14a, unreacted fly ash was observed in the geopolymer binder. Ouda and Gharieb [45] indicated that these unreacted particles reduced the compressive strength of the geopolymer structure. Additionally, microcracks were observed, which were probably caused by the flexural strength testing of the sample (Figure 14b). Numerous pores were also noticed in the geopolymer mortar, which resulted in a relatively high absorbability of the composite. Figure 15a,b show the interphase transition zone (ITZ) between the geopolymer binder and the hemp. This zone has a very important influence on the mechanical properties of geopolymer concrete. This ITZ is quite tight considering the presence of organic material, while the geopolymer mortar partially penetrates the structure of the hemp. However, grains of unreacted FA accumulate in this zone. This is probably due to the fact that hemp a with very high water absorption sucks the water contained in the alkali-activator solution, inhibiting the polymerization process. SEM observations reveal the very porous structure of hemp shives consisting of numerous hollow tubes connected to each other. Their diameter is irregular (Figure 16a,b). Such structure explains both its low bulk density and its high water absorption.

## 4. Conclusions

The paper presents the results of research on the possibility of replacing natural sand in geopolymer mortar with recycled fine and hemp. For this purpose, two research experiments were conducted. Based on the research results, it was found that:

- The recycled fine that is generated as waste in the production of high-quality recycled concrete aggregate, and its amount may constitute up to 60% of the final product. The fines have proved to be a valuable material with high binding properties;
- It can be a valuable substitute for natural sand, which is a non-renewable material. The substitution of 30% by mass of RF as a replacement for natural sand in the alkali-activated mortar increased its flexural and compressive strength by 9% and 26%, respectively;
- RF, as a result of heat treatment, is rich in CaO and SiO_2_ (resulting from the dehydration mainly of Ettringite, Ca(OH)_2_, CSH gel, and Ettringite and Ca(OH)_2_) can favor the formation of new CSH gel in the geopolymer mortar;
- A slight decrease in the absorbability of the mortar was due to the formation of new CSH gel, which produced a compact micro-structure with lower porosity;
- Hemp is a fast-growing renewable material. Its inclusion as a sand substitute caused a decline in the strength properties of geopolymer mortar but with 2.5% substitution (by volume) as a sand replacement. The 40 MPa of compressive strength and 6.3 MPa of flexural strength was obtained;
- Contrastingly, an increase in the water absorption of the geopolymer composite was observed to be up to 10.2% due to the hemp, which due to its structure, has very high absorbability. This means that the hemp must be chemically coated/impregnated prior to being used in the composite to prevent it from moisture suction.

Test results open up the possibility for further research involving the use of recycled fines and hemp as a replacement for part of the natural sand in geopolymer mortars. It seems to be an ecological solution, and geopolymers mortars produced from RF and hemp may prove to be an alternative to cement composites in the future. In addition, the presence of hemp fiber in geopolymer mortars reduces its carbon footprint, which has been confirmed by other researchers [37].

## Figures and Tables

**Table 1:** Chemical composition of fly ash, granulated blast furnace slag, hydrated lime, and recycled fines.

Composition	CaO	Fe_x_O_y_	SiO_2_	Al_2_O_3_	MgO	SO_3_	Na_2_O	K_2_O	LOI	Other Oxides
FA	2.14	4.97	54.6	25.3	1.8	0.37	0.84	2.8	4.37	2.81
GGBS	42.28	1.32	39.04	7.07	6.35	0.73	0.49	0.36	-	2.36
Hyd.Lime	51.01	0.38	3.92	2.74	0.28	-	-	0.04	41.56	0.07
RF	17.40	3.10	58.0	5.9	1.59	0.58	0.95	1.49	10.37	0.62

**Table 2:** The main properties of the hemp.

Properties	Bulk Density in a Loose State, g/cm^3^	Bulk Density in a Compacted State, g/cm^3^	Humidity, %	Water Absorption, %
Hemp	1.17	1.30	1.60	401.94

**Table 3:** The main properties of recycled fines (RF).

Properties	Bulk Density in a Loose State, g/cm^3^	Bulk Density in a Compacted State, g/cm^3^	Skeletal Density, g/cm^3^
Recycled fines	1.05	1.33	2.67

**Table 4:** The results of the elemental composition analysis of recycled fines.

Element	C	O	Al	Si	Ca
Weight (%)	7.6	41.7	0.7	6.9	43.2
Atomic (%)	13.7	56.9	0.5	5.3	23.5

**Table 5:** The percent content of recycled fines as sand replacement.

X	Contents of recycled fines 0–0.125 mm	0%	10%	20%	30%	replacement of standard sand

**Table 6:** The composition of the geopolymer mortars based on the content of recycled fines (on 3 samples 40 mm × 40 mm × 160 mm).

Component	Series			
	0	10%	20%	30%
Fly ash, g	450	450	450	450
Activator, g	270	270	270	270
Standard sand, g	1350	1215	1080	945
Activator/FA ratio	0.60	0.60	0.60	0.60
Recycled fines, g	0	135	270	405

**Table 7:** Variables in the experimental plan.

*X*_1_	Amount of hemp, % of sand by volume	0	2.5	5
*X*_2_	Amount of lime, % of FA	0	2	4

**Table 8:** Experimental plan.

Series No.	Variables	
	*X*_1_, %	*X*_2_, %
1	0	0
2	0	2
3	0	4
4	2.5	0
5	2.5	2
6	2.5	4
7	5	0
8	5	2
9	5	4

**Table 9:** Composition of geopolymer mortar on 3 samples 40 mm × 40 mm × 160 mm.

Series	Fly ash, g	GGBS, g	Activator 8 M, g	Sand, g	Hemp, g	Activator for Hemp, g	Lime, g
1	300	150	270	1392.5	0	0	0
2	294	150	270	1392.5	0	0	6
3	288	150	270	1392.5	0	0	12
4	300	150	270	1357.6	17.1	25.0	0
5	294	150	270	1357.6	17.1	25.0	6
6	288	150	270	1357.6	17.1	25.0	12
7	300	150	270	1322.8	34.2	50.0	0
8	294	150	270	1322.8	34.2	50.0	6
9	288	150	270	1322.8	34.2	50.0	12
